# Poisoning with Pie Plant

**Published:** 1850-09

**Authors:** 


*Poisoning with Pie Plant.*—The Northern Lancet states, that a family in Keeseville, N. Y., were taken alarmingly ill shortly after eating, as *greens*, the leaves of common Rhubarb, or pie-plant. A servant in the family died the following day; all the others recovered.

In the first Vol. of this Journal an instance is given in which a whole family were made ill from the same cause. Physicians should disseminate the liability of serious consequences from the free use of this plant.

